# Research on Radio Altimetry in Urban Environments Based on Electromagnetic Simulation Echo Modeling Technology

**DOI:** 10.3390/s26061932

**Published:** 2026-03-19

**Authors:** Jian Xiong, Xin Xie, Xujun Guan, Yunye Xu, Chao Li

**Affiliations:** 1 Naval University of Engineering, Wuhan 430033, China; 2 Unit 91091, Sanya 572011, China; 3 Unit 91515, Sanya 572000, China

**Keywords:** radar altimetry, shooting and bouncing rays (SBR), echo simulation, multipath propagation, urban environment

## Abstract

As the low-altitude economy develops rapidly, precise radar altimetry is crucial for ensuring the safety and reliability of drone flights. In the context of urban radio detection, the presence of numerous buildings and ground surfaces gives rise to electromagnetic wave multipath propagation. This objective factor gives rise to errors in radar altimetry. Existing channel models often lack the intricate details required to accurately quantify multipath error mechanisms in kilometer-scale complex electromagnetic environments. Therefore, there is an urgent need for a high-fidelity simulation framework. The present study has put forward a pioneering approach to radio altimetry simulation and accuracy assessment in intricate urban environments. The objective of this study is to investigate the impact of multipath propagation on radar altimetry precision. The present study has proposed a novel integration of radar altimetry simulation with kilometre-scale urban electromagnetic simulation models. The simulation of echo signals has been achieved through the utilization of the shooting and bouncing rays (SBR) method and inverse fast Fourier transform (IFFT). A comparative analysis has been conducted based on ranging results from radar systems for different urban models, thereby enabling a mechanism analysis of factors affecting radar altimetry. The study has demonstrated that increased building density and height, along with reduced elevation angles during altimetry, exacerbate ranging errors.

## 1. Introduction

In the contemporary era, radar altimetry has become a pivotal instrument in a multitude of pivotal domains. These domains include military applications such as missile guidance and high-altitude reconnaissance, as well as civilian sectors such as environmental monitoring, resource management, and disaster prevention. The fundamental principle underlying this process entails the precise measurement of the time required for radar waves to travel from the platform to the ground surface and vice versa. This temporal measurement is then utilized to calculate the vertical distance or slant range from the radar antenna to the surface. Consequently, characteristics such as the delay of the echo signal, signal-to-noise ratio, and bandwidth directly influence the accuracy of radar ranging. A multitude of factors have been identified as contributing to errors in radar echo detection, including clutter, multipath effects, and target characteristics. In the context of urban radio detection, the presence of numerous buildings and ground surfaces gives rise to the phenomenon of electromagnetic waves propagating via multipath reflection. This objective factor introduces errors in radar altimetry. The employment of electromagnetic simulation technology in this study facilitates the accurate modelling of multipath echoes in urban environments, thus enabling a quantitative analysis of the impact of multipath propagation on radar altimetry accuracy within complex urban settings.

In order to address the demands of engineering practice, numerous scholars have developed radio echo models employing various methods tailored to different scenarios. The objective of these models is to enhance and optimize the performance of radio detection systems. In the context of echo simulation modeling research, a range of methodologies have been put forth to address the varied demands of different scenarios. Pan et al. employed a target electromagnetic scattering center modeling approach based on dynamic radar cross section (RCS) to achieve highly realistic echo synthesis for moving targets, effectively describing the dynamic electromagnetic scattering characteristics of the target [1]. Wang’s approach entailed the integration of convolutional modeling with two-dimensional fast Fourier transform (FFT) acceleration in echo construction, thereby effectively addressing the computational intensity and time-consuming concerns associated with generating real-aperture radar echoes for Earth observation, super-resolution imaging, and target search [2]. It is well established that noise and clutter are typically significant factors that affect echo characteristics. Addressing wind turbine interference in radar target detection, Zhang developed a wind farm echo simulation model for radar scanning modes based on a scatter point superposition model combined with geometric optics analysis. This theoretical framework provides a foundation for the identification and suppression of wind farm clutter, as well as the design of radar system anti-interference capabilities [3]. In order to confront the challenge of modeling echo signals affected by tropospheric waveguide propagation—which alters signal amplitude, phase, and energy distribution—Hou et al. from Xidian University successfully established an over-the-horizon radar echo signal model integrating atmospheric waveguide propagation with target composite electromagnetic scattering. This objective was accomplished by integrating vector three-dimensional parabolic equations, ray-bouncing methods, and scattering center models [4]. Additionally, addressing the distortion of shoreline echo signals in ship navigation radars, Han systematically simulated the distortion characteristics of shoreline echoes under terrain and propagation effects by integrating a digital elevation model [5]. Research on RCS modelling and characterisation can be divided into two main categories: deterministic modelling and statistical modelling. Statistical methods treat RCS as a random variable, employing probability density functions to describe its fluctuation characteristics. Measurement studies by Azim et al. on indoor factory targets within the 25–28 GHz band demonstrate that the log-normal distribution effectively characterises the statistical RCS properties of moving targets. The results show good agreement with the 3rd generation partnership project (3GPP) integrated sensing and communication (ISAC) channel modelling standard. This research provides key RCS parameters for typical indoor factory targets that are not explicitly defined in the 3GPP standard [6]. Deterministic methods can accurately compute the RCS of a target under specific conditions using electromagnetic field theory. These methods are suitable for scenarios involving precise 3D models and can effectively capture the effects of multiple reflections and diffraction between buildings in urban environments. Buddendick et al. used the shooting and bouncing rays (SBR) method combined with single-station-to-dual-station equivalence principles to efficiently simulate the RCS of complex, large targets at multiple angles using a single radar station [7].

In complex electromagnetic environments, the multipath echo effect caused by buildings and terrain is a significant factor that degrades radio ranging performance. This issue poses a significant challenge to target detection and has been the subject of extensive research. In order to address the issue of false target detection caused by multipath propagation in automotive radar systems, Liu C. et al. proposed a multipath propagation model and mathematical formula for damaged echoes. This approach has been demonstrated to effectively eliminate ghost targets in automotive radar during multi-target scenarios, thereby enhancing ranging accuracy [8]. Shishanov et al. proposed a detection algorithm based on comparing the angular consistency between physical and virtual antenna arrays. This effectively distinguishes real targets from ghost targets in radar echoes, thereby providing a solution to enhance the reliability of advanced driver assistance systems [9]. Ming and Li’s study addressed the issue of environmental clutter interference affecting low-altitude, slow-moving small target echoes in urban environments. The integration of RELAX clutter suppression with adaptive constant false alarm rate (CFAR) detection has been demonstrated to enhance target detectability to a substantial degree [10]. In order to address the issue of multipath interference in global system for mobile communications (GSM) passive radar within dense urban settings, Krysik incorporated a constant modulus algorithm with a freeze-adaptive mechanism, thereby effectively suppressing multipath interference in the reference channel [11]. Furthermore, Arriba-Ruiz employed time-reverse synthetic aperture radar (TR-SAR) to effectively mitigate multipath effects while substantially enhancing target resolution [12]. Knapskog optimized synthetic aperture radar (SAR) imaging parameters, including incidence angle and direction, to reduce image blurring caused by sea surface multipath in port scenarios, providing research directions for optimizing SAR image acquisition strategies in port surveillance [13]. Sinha and Bar-Shalom proposed a maximum likelihood estimation-based angle extraction method to address radar azimuth measurement errors for low-elevation targets in sea-surface multipath environments. By jointly processing the sum and difference channel signals of single-pulse radar, they effectively reduced azimuth measurement bias and significantly decreased the bias and root mean square error in target height estimation [14].

As previously mentioned, the extant literature elucidates the significant impact of factors such as clutter, multipath effects, and target characteristics on radar detection performance. With regard to radio echo simulation and analysis, references [1,2,3,4,5] propose distinct echo simulation methods under various scenarios, primarily focusing on enhancing simulation fidelity. The literature [6,7] focuses on the modeling and characterization of RCS properties. The literature [10,11] introduces algorithms for suppressing clutter and interference in echo simulation within multipath scenarios. The prevailing focus of contemporary analyses of detection accuracy in multipath environments pertains to applications such as automotive radar detection in urban canyon effects and radar measurements under sea surface multipath conditions. A paucity of precise models and high-accuracy analyses persists for large-scale, complex scenarios, such as urban environments [8,9,10,11,12,13,14]. In contemporary urban environments, a plethora of structures and trees not only cause radar signal occlusion, diffraction, and multipath effects but also introduce clutter interference. This results in substantial alterations to target echo characteristics, consequently leading to inaccurate detection and recognition. Consequently, the implementation of radar altimetry in complex urban settings poses significant challenges. Consequently, the evaluation of radar altimetry’s precision in urban environments is of considerable significance.

**Figure 1:**
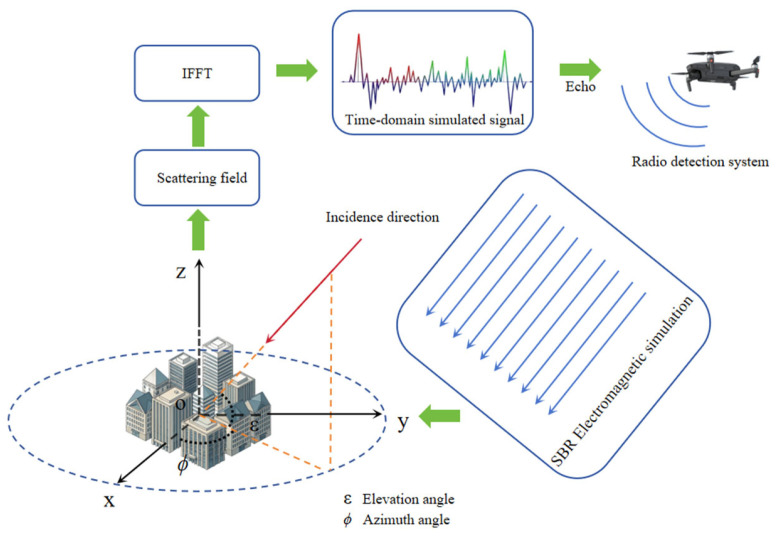
Echo signal simulation and radar ranging scenario diagram. Note: The figure illustrates a coordinate system that has been established with the geometric centre of the city model’s ground surface designated as its origin. In this context, ϕ denotes the azimuth angle, defined as the angle between the projection of the incident vector onto the *xoy* plane and the positive direction of the *x*-axis. ε is employed to denote the elevation angle, which is defined as the angle between the incident vector and the *xoy* plane.

The present study is distinct from previous literature in that it investigates the impact of complex urban radio echo multipath propagation on radar altimetry accuracy. The innovative integration of radar altimetry with electromagnetic simulation of large-scale targets proposes a novel method for radio altimetry simulation and accuracy analysis in complex urban environments. This approach provides novel insights for subsequent research in related fields. Furthermore, the study employs the shooting and bouncing rays (SBR) method for echo simulation, effectively modeling the complex propagation effects of electromagnetic waves in large urban environments while significantly enhancing computational and simulation efficiency. A series of high-frequency electromagnetic simulations were conducted on urban models of kilometer-scale, employing the SBR method. The frequency response data obtained from these simulations were converted into time-domain signals via inverse fast Fourier transform (IFFT), thereby enabling the simulation of radio echo propagation in urban environments. A radar altimetry simulation model has been developed to detect echo signals, evaluate radar altimetry accuracy, and analyze the impact of multipath propagation in urban environments on radar altimetry performance. The basic process is shown in Figure 1.

## 2. Analysis of Electromagnetic Scattering Characteristics in Urban Environments

Initially, electromagnetic characteristic parameters are assigned to the three-dimensional urban model to ensure the authenticity of phenomena such as target reflection and diffraction. Subsequently, the urban model undergoes multi-frequency electromagnetic wave scanning by employing the SBR method. This process yields frequency-domain responses at various locations relative to the reference origin. The multipath propagation of rays within the urban model effectively simulates the multipath propagation effects of radar signals. The data obtained from the electromagnetic simulation undergoes an IFFT to complete the simulation of time-domain echo signals, providing data for subsequent radar altimetry accuracy evaluation.

### 2.1. Construction of an Electromagnetic Simulation Model for Urban Environments Based on the SBR Method

For solving electromagnetic scattering problems involving large-scale targets with complex electromagnetic wave propagation effects, such as urban models, high-frequency methods are typically chosen due to their faster computation speed, lower memory consumption, and high computational efficiency [15,16,17,18]. Among various high-frequency methods, the SBR method has evolved through integration with theories like geometrical theory of diffraction (GTD) and uniform theory of diffraction (UTD). This advancement significantly enhances the accuracy and efficiency of electromagnetic simulations, establishing SBR method as one of the most mainstream algorithms in contemporary electromagnetic simulation computing [19]. The SBR method simulates a large number of rays originating from a source point. Based on geometric optics (GO), it assumes electromagnetic waves propagate along straight paths and undergo reflection and diffraction effects upon encountering target surfaces. The path of each ray and its derived sub-rays is traced until the ray energy falls below a threshold or reaches a receiver point. Finally, the required electromagnetic information is obtained through integration calculations [20].

The electromagnetic model is now being constructed:

#### 2.1.1. Ray Tube Generation for Urban Environments

**Figure 2:**
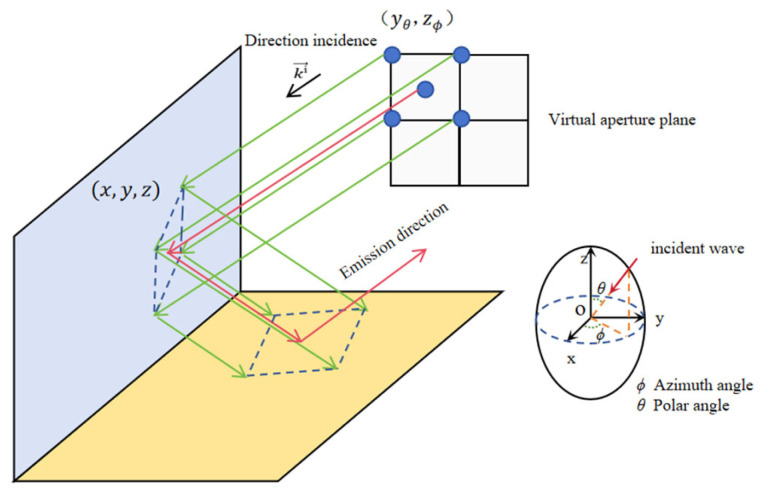
Ray tube generation. Note: Figure 2 illustrates the construction of the virtual aperture and the process of ray tube subdivision.

Ray tube generation involves virtual aperture construction and ray tube subdivision. Typically, ray tubes of a specified density simulate incident electromagnetic waves, propagating according to GO theory. Through one or multiple reflections and refractions, rays ultimately reach the receiver position. As shown in Figure 2, this study employs a ray tube structure comprising “one central ray and four corner rays.” This design is crucial for accurately capturing multipath interference caused by complex geometries such as urban canyons and building edges.

The ray tube is generated as shown in Figure 2:

1. Virtual Aperture Construction

In order to ensure that the incident rays cover the entire kilometre-scale urban area, the incident wave is discretised into ray tubes originating from a virtual aperture plane grid. The dimensions of the virtual aperture are determined by projecting onto the target vertices.

The direction of the incident electromagnetic wave is defined as follows:

(1) $$k_{i}\;=\;-2\pi {\left[\begin{array}{ccc} sin\theta \cos\phi & sin\theta sin\phi & \cos\theta \end{array}\right]}^{T}/\lambda$$

As shown in Figure 2, θ is the polar angle (θ = 90° − ε, measured from the positive *z*-axis to the radial direction); ϕ is the azimuth angle (measured in the *xoy* plane from the positive *x*-axis to the projection of the radial direction); λ is the wavelength of the incident wave.

Select a plane perpendicular to the electromagnetic wave incidence direction and located at a distance d from the origin of the 3D coordinate system to construct a virtual aperture. For kilometer-scale urban areas, to ensure wavefront curvature error remains below 5% while minimizing redundant rays, d is set to 1.5 times the diameter of the city model’s bounding sphere. Iterate through all vertices of the target’s triangular faces, projecting each vertex onto the virtual aperture plane. This transforms the vertex’s global coordinates $\left(x,\;y,\;z\right)$ into projected coordinates $\left(y_{\theta },z_{\phi }\right)$ on the virtual aperture plane. The coordinate transformation formula is as follows:

(2) $$\left[\begin{array}{c} y_{\theta }\\ z_{\phi } \end{array}\right]=\;\left[\begin{array}{ccc} \cos\theta \cos\phi & \cos\theta sin\phi & -\sin\theta \\ -\sin\phi & \cos\phi & 0 \end{array}\right]\left[\begin{array}{c} x\\ y\\ z \end{array}\right]$$

(3) $$\left[\begin{array}{c} x\\ y\\ z \end{array}\right]=F^{T}\left[\begin{array}{c} d\\ y_{\theta }\\ z_{\phi } \end{array}\right]$$

(4) $$F=\left[\begin{array}{ccc} \sin\theta \cos\phi & \sin\theta \sin\phi \; & \cos\theta \\ \cos\theta \cos\phi & \cos\theta \sin\phi & -\sin\theta \\ -\sin\phi & \cos\phi & 0 \end{array}\right]$$

The unit vectors are defined as follows: $\left[\begin{array}{ccc} \sin\theta \cos\phi & \sin\theta \sin\phi \; & \cos\theta \end{array}\right]$ represents the unit vector in the incident direction, perpendicular to the virtual aperture plane; $\left[\begin{array}{ccc} \cos\theta \cos\phi & \cos\theta \sin\phi & -\sin\theta \end{array}\right]$ represents the unit vector in the polar angle direction, lying within the incident plane; $\left[\begin{array}{ccc} -\sin\phi & \cos\phi & 0 \end{array}\right]$ represents the unit vector in the azimuth direction, perpendicular to the incident plane.

It is possible to obtain the projection coordinates $\left(y_{\theta },z_{\phi }\right)$ of different face vertices in the target 3D city model, along with the position of the virtual aperture, through the above process. The coverage area of the virtual aperture can then be defined as: $\left[y_{\theta \min},y_{\theta \max}\right]$ and $\left[z_{\phi \min},z_{\phi \max}\right]$.

2. Ray Tube Subdivision

To ensure the accuracy and computational efficiency of the SBR method, it is customary to subdivide ray tubes within the ranges $\left[y_{\theta \min},y_{\theta \max}\right]$ and $\left[z_{\phi \min},z_{\phi \max}\right]$ with a step size $\delta_{r}$ less than or equal to 1/10 of the incident wavelength. This configuration ensures the presence of at least 10 ray tubes per wavelength, thereby facilitating the precise capture of phase variations induced by multipath interference in urban environments.

The total number of emitted ray tubes is $N_{\mathrm{tube}}$, and the total number of rays is $n_{\mathrm{total}}$:

(5) $$N_{\mathrm{tube}}\;={\;N}_{\theta }\;\times {\;N}_{\phi }$$

(6) $$N_{\theta }=\left\lceil \frac{1}{\delta_{r}}(y_{\theta \max}\;-\;y_{\theta \min})\right\rceil ,N_{\phi }=\left\lceil \frac{1}{\delta_{r}}(z_{\phi \max}\;-{\;z}_{\phi \min})\right\rceil$$

(7) $$n_{\mathrm{total}\;}\;=5\times N_{tube}$$

$N_{\theta }$ and $N_{\phi }$ a245re rounded up, respectively.

Let the ray set in the ray tube be denoted as $\left\{L_{i,j,k}|i\;\in \;[0,n_{\theta }-1],\;j\;\in \;[0,n_{\phi }-1],\;k\;\in \;\left[1,5\right]\right\}$, where *i* and *j* represent the indices of rays in the $y_{\theta }$ and $z_{\phi }$ directions, respectively. When the ray is the central ray, *k* = 1, used to carry phase, amplitude, and polarization vector information. When the ray is one of the four corner rays, *k* = 2, 3, 4, 5, respectively, for geometric path tracing. The coordinates of the ray on the virtual aperture plane are:

(8) $$\begin{array}{c} y_{i}\;={\;y}_{\theta \min}\;+\;(i\;+\;Q_{k,1})\delta_{r}\\ z_{j}\;={\;z}_{\phi \min}\;+\;(j\;+{\;Q}_{k,2})\delta_{r} \end{array}$$

(9) $$Q=\left[\begin{array}{cc} 0.5 & 0.5\\ 0 & 0\\ \begin{array}{c} 1\\ \begin{array}{c} 1\\ 0 \end{array} \end{array} & \begin{array}{c} 0\\ \begin{array}{c} 1\\ 1 \end{array} \end{array} \end{array}\right]$$

The first row of matrix Q represents the indices of the central rays, while the remaining rows represent the positional indices of the angular rays.

#### 2.1.2. Ray Tracing

**Figure 3:**
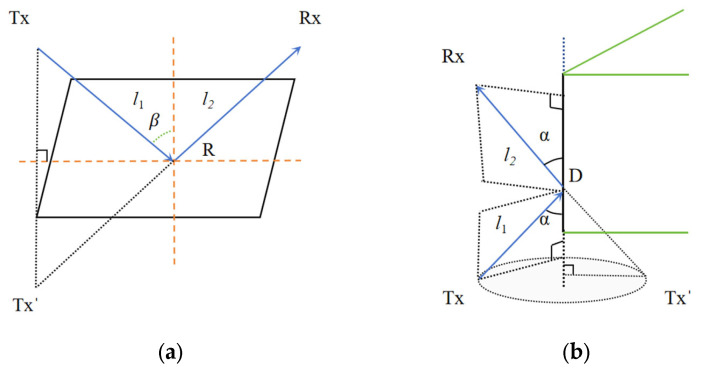
(**a**) Schematic diagram of reflection. (**b**) Schematic diagram of diffraction.

Rays emitted from the virtual aperture plane into the urban model region undergo one or multiple reflections and diffractions. Ray tracing based on GO theory is required to determine the propagation paths of these rays. Electromagnetic field theory is then applied to calculate field strength and other parameters at target locations along these paths, enabling precise modelling within the specified region [21]. Nevertheless, the GO theory is unable to provide an explanation for diffraction phenomena that occur at model edges, vertices, and shadow regions. Consequently, in such scenarios, the combination of GO theory with the UTD theory is imperative to resolve these issues. The various effects of ray propagation are illustrated in Figure 3:

Let the incident ray be T(x):

(10) T(x) = Tx + sx, x ≥ 0

where *s* is the direction vector of the incident ray; T(x) is the position vector of the incident point; *x* is the propagation distance parameter; β is the angle of reflection; $l_{1}$ is the distance from the incident point to the reflected point *R*.

When $x\;=\;l_{1}$, point $T_{1}\;=\;T\left(l_{1}\right)$ intersects the surface element within the target plane and undergoes reflection. The ray must be tracked continuously until it exits the target. Approximated by GO theory, the reflected ray satisfies Fresnel principle. Let *n* denote the unit normal vector of the intersecting surface element. Then the direction vector of the reflected ray is:

(11) $$s_{1}\;=\;s\;–\;2n\left(s\cdot n\right)$$

The reflected ray is obtained as $R\left(x\right)$:

(12) $$R(x)\;=\;\mathrm{Rx}\;+{\;s}_{1}x,\;x\;\geq \;0$$

where $s_{1}$ is the direction vector of the reflected ray; *R* is the position of the reflection point; *Rx* is the position vector of the target point; $l_{2}$ is the distance from the target point to the reflection point [19].

After reflection occurs, the amplitude, phase, and polarization of the ray tube require further calculation.

The incident electric field intensity is decomposed into a horizontally polarized component $E_{∥}$ and a vertically polarized component $E_{⊥}$. The unit vector of the vertically polarized component is $e_{⊥}\;=\;\frac{s\;\times \;n}{\left|s\;\times \;n\right|}$, while the unit vector of the horizontally polarized component is $e_{∥}=\frac{s\;\times \;e_{⊥}}{\left|s\;\times \;e_{⊥}\right|}$. The unit vector of the horizontally polarized component after reflection is $e_{∥}^{r}=\frac{s_{1\;}\times \;e_{⊥}}{\left|s_{1}\;\times \;e_{⊥}\right|}$. Within the initial ray tube bundle generated on the virtual aperture plane, all rays share the initial polarization state defined by the central ray. During propagation, the polarization state of the ray tube is iteratively updated as the central ray propagates. If the ray tube splits, the generated sub-ray tubes inherit the latest polarization vector of the parent ray tube’s central ray at the split point.

The field strength can be determined using the following equation:

(13) $$\left\{\begin{array}{c} E_{i}\;={\;E}_{⊥}e_{⊥}\;+\;E_{∥}e_{∥}\\ {\;E}_{r}\;={\;E}_{⊥}R_{⊥}A(l_{1},l_{2})e^{-\mathrm{jk}(l_{1}\;+\;l_{2})}e_{⊥}+E_{∥}R_{∥}A(l_{1},l_{2})e^{-\mathrm{jk}(l_{1}\;+{\;l}_{2})}e_{∥}^{r} \end{array}\right.$$

(14) $$R_{⊥\;}=\frac{\cos\theta -\sqrt{\epsilon_{r}}-\sin^{2}\theta }{\cos\theta +\sqrt{\epsilon_{r}}-\sin^{2}\theta }$$

(15) $$R_{∥}=\frac{\sqrt{\epsilon_{r}-\sin^{2}\theta -\epsilon_{r}\cos\theta }}{\sqrt{\epsilon_{r}-\sin^{2}\theta +\epsilon_{r}\cos\theta }}$$

(16) $$A(l_{1},l_{2})=\frac{l_{1}}{l_{1}+l_{2}}$$

where $E_{i}$ denotes the incident electric field intensity; $E_{r}$ denotes the reflected electric field intensity; $R_{⊥}$ represents the reflection coefficient of the vertically polarized component at the reflecting surface; $R_{∥}$ represents the reflection coefficient of the horizontally polarized component at the reflecting surface; *k* denotes the wave number ($k=\frac{2\pi }{\lambda }$); $\epsilon_{r}$ denotes the dielectric constant of the surface material; $A(l_{1},l_{2})$ denotes the divergence factor for plane reflection [22].

As illustrated in Figure 3, when diffraction occurs in the incident rays, the diffraction field at the edge is calculated using UTD theory. The diffraction field can be computed using the following formula:

(17) $${\;E}_{d}\;=\;E_{⊥}D_{⊥}A_{d}e^{-{\mathrm{jkl}}_{2}}e_{d⊥}\;+\;E_{∥}D_{∥}A_{d}e^{-{\mathrm{jkl}}_{2}}e_{d∥}$$

(18) $$A_{d}(l_{1},l_{2})=\sqrt{\frac{l_{1}}{{l_{2}(l}_{1}+l_{2})}\;}$$

where $A_{d}$ denotes the diffraction divergence factor; $D_{⊥}$ and $D_{∥}$ represent the diffraction coefficients; $l_{1}$ is the distance from the incident point to the diffraction point; $l_{2}$ is the distance from the target point to the diffraction point; $e_{d⊥}$ denotes the unit vector in the vertical polarization direction of the electric field; $e_{d∥}$ denotes the unit vector in the horizontal polarization direction of the electric field [23,24].

In order to accurately capture the effects of multipath propagation in urban environments while maintaining computational efficiency, this study uses the maximum number of reflections as the main criterion for terminating ray tracing. Given that densely distributed urban structures can cause electromagnetic waves to undergo multiple reflections, the maximum reflection count is set to 20. In urban environments, the energy carried by rays typically decays to levels far below the noise floor of typical radar receivers after 20 reflections. Therefore, this setting captures the primary multipath components that cause altimetry errors, thereby ensuring the accuracy of the simulation results [22,23,24].

#### 2.1.3. Field Strength Integration

Subsequent to the completion of ray tracing, the far-field electric field strength must be calculated. It is evident that a solitary ray tube exerts a negligible influence on the field strength, thereby rendering it virtually inconsequential for the purposes of electromagnetic analysis. The integration of the dispersed field of the urban model in accordance with the principles of physical optics (PO) results in the determination of the ultimate far-field electric field strength.

**Figure 4:**
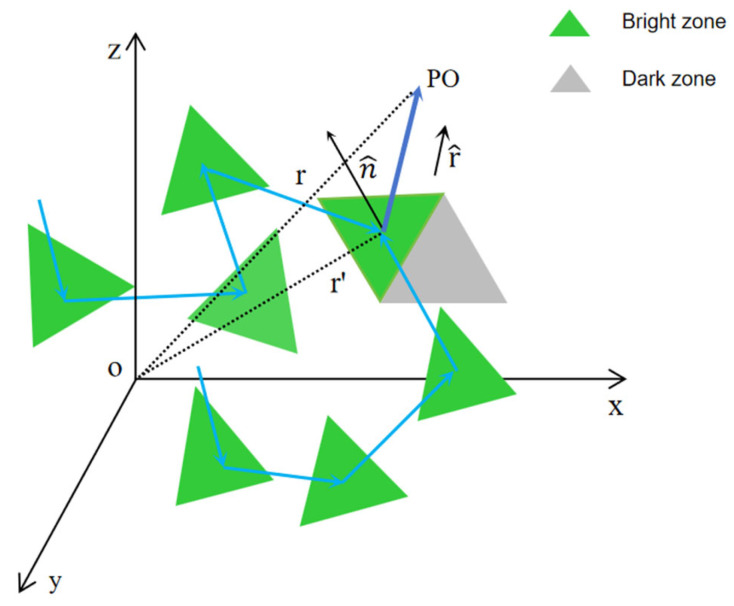
Schematic diagram of PO integration. Note: the field strength integration at a distance *r* after the rays propagate through multiple mesh elements.

In intricate urban environments, the complex geometry of buildings leads to multiple reflections and diffraction during ray propagation, significantly increasing computational complexity. As shown in Figure 4, in the calculation of the field strength integral, building surfaces are discretized into triangular or quadrilateral mesh elements that function as PO integration cells. The induced field strength generated by surface currents on each illuminated surface is computed, and the scattering field is subsequently obtained through integration [25].

For non-conductive targets, no actual conductive current exists on the surface. This study employs the equivalence principle to characterize the scattering effects generated by internal polarization and magnetization within the target using equivalent current *J* and equivalent magnetic flux *M*:

(19) $$J\;=\;n\;\times \;H_{\mathrm{total}}$$

(20) $$=\frac{1}{Z_{0}}[(1\;–{\;R}_{⊥})E_{⊥}\cos\theta e_{⊥}+(1+{\;R}_{∥})E_{∥}(n\;\times \;e_{⊥})]$$

(21) $$M={\;E}_{\mathrm{total}}\;\times \;n$$

(22) $$=(1\;–{\;R}_{∥})E_{∥}\cos\theta e_{⊥}\;-\;(1+R_{⊥})E_{⊥}(n\;\times {\;e}_{⊥})$$

The scattered field $E_{r}$ at a distance *r* can be obtained using the Stratton-Chu formula:

(23) $$E_{r\;}=\;\frac{jk_{0}e^{-jk_{0}r}}{4\pi r}\int_{S}\hat{s}\;\times \;(Z_{0}\hat{s}\;\times \;J\;+\;M)e^{jk_{0}\hat{s}\cdot r^{\prime }}{\mathrm{dS}}^{\prime }$$

For electromagnetic simulation calculations of perfectly electrically conducting (PEC) materials, the target scattering field can be obtained using the Stratton-Chu formula [19]:

(24) $$H_{\mathrm{total}}\;=\;2H_{i},\;M\;=0$$

(25) $$E_{r}=\frac{jk_{0}e^{-jk_{0}r}}{4\pi r}\int_{S}\hat{s}\;\times \;(Z_{0}\hat{s}\;\times \;J)e^{jk_{0}\hat{s}\cdot r^{\prime }}{\mathrm{dS}}^{\prime }$$

where $k_{0}$ is the wave number; $Z_{0}$ is the free-space wave impedance; *n* is the unit normal vector to the target surface; $H_{\mathrm{total}}$ and $E_{\mathrm{total}}$ are the total magnetic and electric fields on the target surface; $H_{i}$ is the incident magnetic field; $\hat{s}$ is the scattering direction vector; $r^{\prime }$ is the position vector of the target surface; *S* is the area of the bright region [26,27,28,29].

### 2.2. Electromagnetic Simulation Calculation

#### 2.2.1. 3D City Model

**Figure 5:**
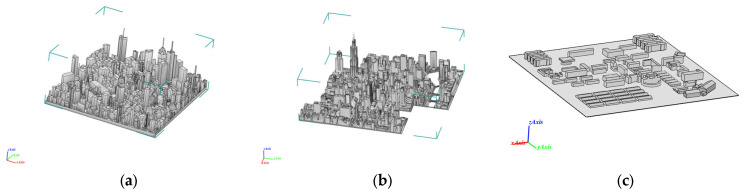
(**a**) New York City neighborhood model (Model A). (**b**) Downtown Chicago model (Model B). (**c**)Wuhan neighborhood model (Model C).

Electromagnetic simulations were conducted on various urban models employing the SBR method. Two distinct types of urban models were selected for comparative analysis. One typology features densely distributed buildings, with many exceeding 100 m in height. Illustrative examples include the simulated Manhattan district in New York City (Model A) and downtown Chicago (Model B), as shown in Figure 5. Another typology features low building density, with structures generally not exceeding 50 m in height. An example of this is the Wuhan neighborhood model (Model C), constructed in this study.

The city model diagram is shown in Figure 5:

#### 2.2.2. Electromagnetic Parameters

In the aforementioned urban scenarios, buildings are predominantly constructed from concrete materials, with most areas of the urban environment covered by large structures. Consequently, for large-scale urban environments of such kilometer-scale, radio propagation can be approximated as Outdoor-to-Indoor propagation loss (O2I loss). The efficacy of this approximation is demonstrated by its application in large urban scenarios, as evidenced by the conversion of the material transmission loss formula from 3GPP TR 38.901 to obtain its equivalent reflection coefficient and transmission coefficient [29,30,31].

Table 1 shows transmission path loss for different materials in urban environmental settings:

The electromagnetic property parameters of different materials can be converted using the formula in Table 1. Model C exhibits relatively low building density, with primary structures consisting of residential buildings, office buildings, and cafeterias. For such scenarios, to enhance parameter accuracy, the low-loss model within the O2I building penetration loss model must be employed to calculate electromagnetic parameters.

(26) $${\mathrm{PL}}_{\mathrm{tw}}=5\;–\;10\log_{10}\left(0.3\cdot 10^{\frac{-L_{\mathrm{glass}}}{10}}+0.7\cdot 10^{\frac{-L_{\mathrm{concrete}}}{10}}\right)$$

#### 2.2.3. Simulation Calculation

**Figure 6:**
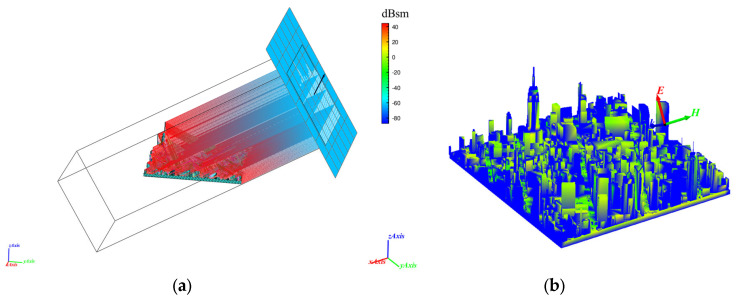
(**a**) The process of ray incidence and reflection during electromagnetic simulation. (**b**) RCS hotspot distribution in the city model generated at simulation conclusion.

In order to reveal the influence mechanism of urban geometric characteristics on radar altimetry accuracy in a systematic manner, this study conducts multi-parameter electromagnetic simulations for the model ABC. As illustrated by Figure 6, the electromagnetic simulation process for the city and the resulting urban electromagnetic scattering model after simulation completion can be effectively represented.

Set the incident wave frequency range to 5 GHz–10 GHz, with elevation angles configured at five typical values: 10°, 30°, 50°, 70°, and 90°. This characterizes the complete transition from low-angle incidence to perpendicular incidence. The ray tube step size is set to $\delta_{r}\;\leq \;\lambda /10$, with a value of 3 mm in this study. This ensures at least 10 rays within a single wavelength, enabling accurate capture of phase variations. The cross-sectional area $\Delta s\;={\;\delta }_{r}^{2}\;\approx \;9\;{\mathrm{mm}}^{2}$ is sufficient to resolve scattering differences between adjacent building walls.

Following the simulation, the electric field intensity, phase distribution, and RCS diagrams of the same city model were obtained at different elevation angles.

**Figure 7:**
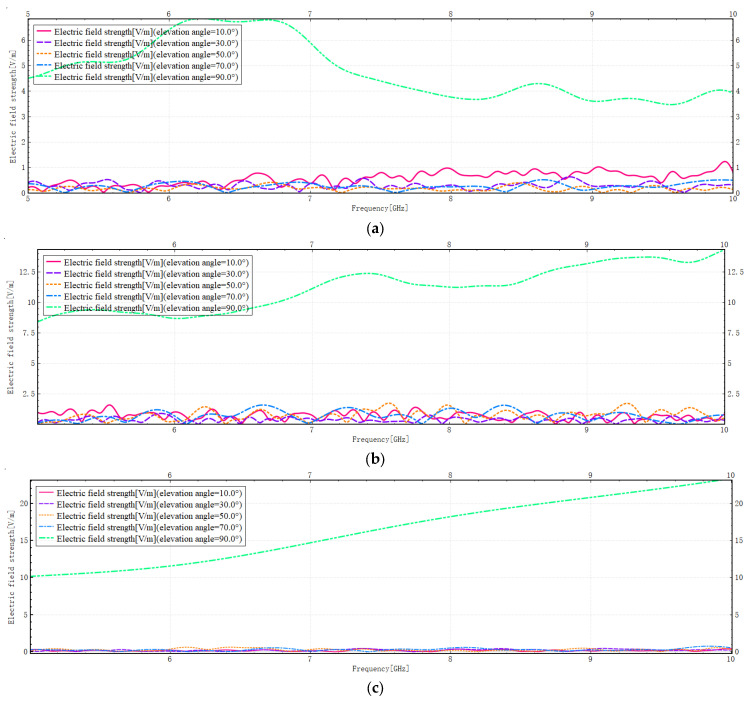
(**a**) Variation of field strength for Model A with frequency. (**b**) Variation of field strength for Model B with frequency. (**c**) Variation of field strength for Model C with frequency.

The field strength distribution at different frequencies is shown in Figure 7:

As illustrated in Figure 7, at elevation angles of 10°, 30°, 50°, and 70°, the electric field strength curves for all three models demonstrate oscillatory behaviour and relatively low field strengths across different frequencies. This finding suggests that multipath propagation occurs at these angles, resulting in substantial interference. Concurrently, the propagation of electromagnetic waves is influenced by architectural impediments and geometric attributes, with the mechanisms of scattering being predominantly characterised by multiple reflections and diffraction. These factors increase propagation paths, causing substantial energy dissipation and maintaining overall low energy levels. At an elevation angle of 90 degrees, the field strength undergoes a substantial increase, and the field strength curve ascends gradually with increasing frequency. This finding suggests that the dominant scattering mechanism at this particular point is specular reflection, which results in the shortest possible propagation path and consequently minimises energy loss caused by multipath effects. A comparison of the three scenarios reveals that, at a 90-degree elevation angle, Model C exhibits a higher field strength amplitude than Models A and B. This phenomenon can be attributed to the sparse building distribution and simpler structural design of Model C, where specular reflection dominates the scattering processes, resulting in propagation that approximates direct transmission. Models A and B feature higher building density and more complex geometric structures, resulting in multiple propagation paths. It is evident that a considerable proportion of multiple reflections and diffraction still occurs, which consequently results in greater energy attenuation and lower field strengths in comparison to Model C. Simultaneously, the diverse propagation paths continue to cause interference phenomena. For instance, Model A exhibits a dip in its curve between 7 GHz and 8 GHz, attributed to destructive interference that reduces energy.

**Figure 8:**
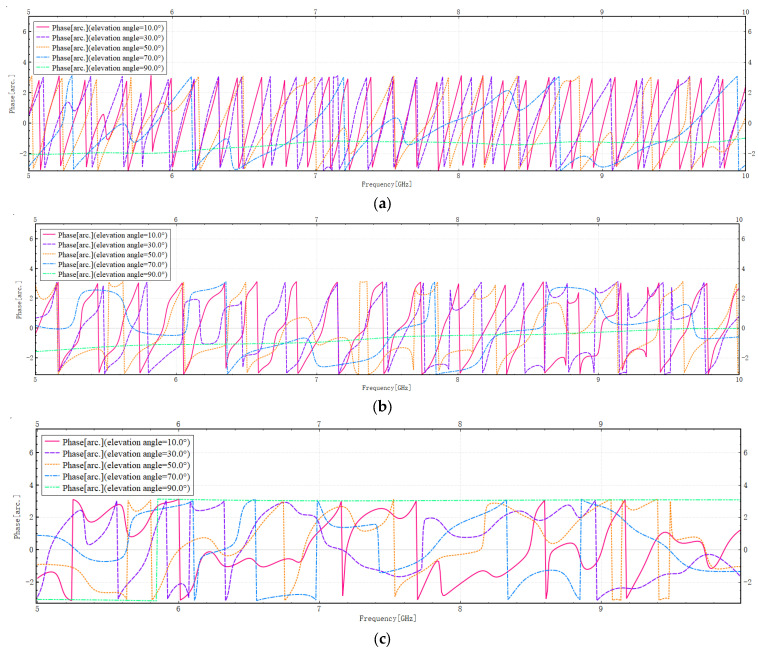
(**a**) Variation of phase for Model A with frequency. (**b**) Variation of phase for Model B with frequency. (**c**) Variation of phase for Model C with frequency.

The phase distribution diagram at different frequencies is shown in Figure 8:

As illustrated by Figure 8, analysis of Model A indicates that at elevation angles of 10°, 30°, 50°, and 70°, the phase demonstrates linear periodic oscillations of the phase winding. Subsequent to the relaxation phase, the phase increases almost linearly with frequency. The distance between the buildings is represented by ∆d. The round-trip path difference between electromagnetic waves incident on two adjacent buildings is therefore given by ΔL = 2Δdsinθ. When the phase difference for one interference cycle is expressed as $\Delta \phi (f)\;=\;2\pi =\frac{2\pi \Delta f}{c}\Delta L$, the frequency Δf of interference cycles in Model A fluctuates minimally. Consequently, the values of ΔL and ∆d remain almost constant. This finding indicates that, despite the dense and tall building distribution of Model A, the structures essentially form a grid pattern with equidistant walls and orderly positioning, thereby creating an equivalent “scattering array” that is both dense and orderly, rather than chaotic. Calculations demonstrate that ΔL exceeds actual block distances, confirming the interference effect arises from multiple reflections causing ordered interference among propagation paths. This analysis aligns with the actual grid layout of Manhattan’s city blocks. At an elevation angle of 90 degrees, electromagnetic waves strike perpendicularly, causing the phase to rise slowly and steadily. Consequently, the interference effect is weak, with the primary propagation path dominated by specular reflection, effectively suppressing multipath effects.

Analysis of Model B reveals that at elevation angles of 10°, 30°, 50°, and 70°, the phase variation demonstrates periodic oscillations similar to Model A. These oscillations are accompanied by significant multipath effects and interference phenomena. However, at elevations of 50° and 70°, the periodicity of phase changes diminishes, interference patterns become less discernible, and interference cycle frequencies become unstable. As demonstrated in the preceding analysis, the configuration of buildings in Model B is characterised by a greater degree of disorder when compared with Model A. The interference cycle frequency in Model B is lower than that in Model A, indicating a reduced building density in Model B. However, given the presence of a multitude of densely packed structures, multipath propagation of electromagnetic waves remains predominant. At an elevation angle of 90 degrees, the phase variation pattern aligns with Model A, where the primary propagation path is dominated by specular reflection, thereby suppressing multipath effects.

A thorough examination of model C reveals that at elevations of 10°, 30°, 50°, and 70°, phase transitions are pronounced without any discernible periodicity or regularity. Furthermore, the phase change rate is lower than that observed in model A and B. This phenomenon can be attributed to the limited number and sparse distribution of buildings, which results in a reduced number of electromagnetic wave scattering paths. Consequently, there is a significant decrease in the number of reflections along the propagation path compared to model ab. Concurrently, the substantial randomness inherent in the distribution of building locations gives rise to frequent disordered random interference. Phase changes exhibit nonlinear and non-periodic variations with frequency shifts, with disordered random interference occurring frequently. At an elevation angle of 90 degrees, the electromagnetic wave propagation path is simplified. Propagation is dominated by specular reflection, with non-direct paths exhibiting extremely weak energy. The multipath effect is effectively mitigated. At this juncture, the frequency-phase diagram approaches a linear configuration more closely than in Models A and B, exhibiting a reduced gradient. This phenomenon is further evidenced by the relatively sparse and simple structure of buildings in Model C, which results in shorter electromagnetic propagation paths, consistent with objective reality.

**Figure 9:**
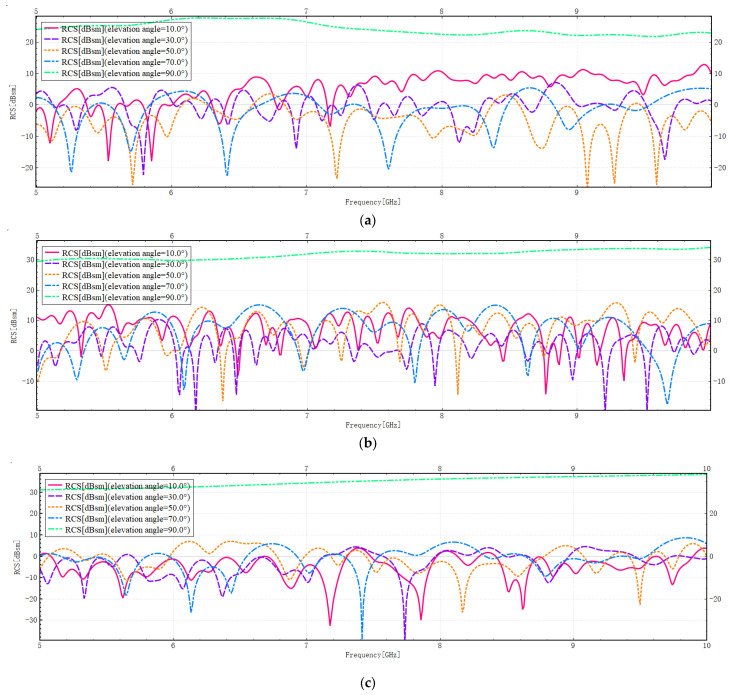
(**a**) Variation of RCS for Model A with frequency. (**b**) Variation of RCS for Model B with frequency. (**c**) Variation of RCS for Model C with frequency.

The RCS distribution at different frequencies is shown in Figure 9:

A detailed analysis of Figure 9 indicates that at elevation angles of 10°, 30°, 50°, and 70°, the RCS images for Models A, B, and C demonstrate oscillatory and unstable states. This outcome is indicative of pronounced multipath propagation effects for all three models, accompanied by significant interference phenomena. At an elevation angle of 90 degrees, the images stabilise as electromagnetic wave propagation is dominated by specular reflection, thereby suppressing interference effects.

The average RCS values for the three models are shown in Table 2:

As shown in Table 2, at low elevation angles, such as 10 and 30 degrees, Model C exhibits the smallest average RCS. This is primarily due to the fact that Model C features sparse and low-rise buildings. At low elevation angles, the equivalent area available for reflecting radar echoes is reduced, resulting in a smaller average RCS. As the elevation angle increases, the average RCS also gradually increases. It is evident that the intricate configuration of the building distribution in models A and B gives rise to the pronounced manifestation of multipath propagation effects. Consequently, their average RCS fluctuates significantly at different elevation angles due to interference effects and energy propagation attenuation. At an elevation angle of 90 degrees, Model C exhibits the largest RCS, followed by Model B, with Model A being the smallest. The main reason for this is that, when electromagnetic waves are incident vertically, the primary source of backscattering is specular reflection from the ground surface. Model C has a sparse, low-rise building layout which exposes most of the ground area directly to the incident waves, creating a large effective reflective surface. In contrast, the high-density, high-rise layout of Models A and B results in significant ground shading by buildings, yielding a smaller effective reflective surface area. Furthermore, due to the complex structures of Models A and B, a certain proportion of multipath propagation still occurs. However, each reflection is accompanied by energy attenuation, and some paths may also generate destructive interference, which further reduces the overall backscatter intensity.

Electromagnetic simulations were conducted on three distinct urban models, with qualitative analysis performed on the curves representing field strength, phase, and RCS. The multipath propagation mechanisms and energy loss distribution of electromagnetic waves were found to be significantly influenced by geometric characteristics such as building density, height, and ground visibility at different elevation angles. This resulted in variations among the simulation outcomes for different models. These disparities in propagation characteristics directly resulted in divergent ranging errors across various scenarios.

It is important to acknowledge that, despite the SBR method employed in the aforementioned simulations being appropriate for urban model calculations, it is not without its limitations. The SBR method is a high-frequency approximation technique; however, it is subject to reduced accuracy when dealing with scattering problems involving electrically small targets. Consequently, the resolution of associated issues can be accomplished through the integration of methodologies such as the method of moments (MOM) and the finite-difference time-domain (FDTD) approach.

## 3. Time-Domain Reconstruction of Echo Signals

### 3.1. Time-Domain Reconstruction Method for Electromagnetic Scattering Signals

This study focuses on analysing static multipath propagation errors caused by urban geometric structures, with the primary aim of evaluating the accuracy of radio altimetry in drone hovering scenarios. In this context, the Doppler shift can be considered negligible and the urban multipath channels can be treated as linear time-invariant (LTI) systems.

Through the utilization of electromagnetic simulation calculations, the electric field strength at equal frequency intervals is derived. The time-domain echo signals from the radio detection system can be equivalently represented by the impulse response derived via IFFT. This method finds application in complex reflection scenarios involving multiple scattering centres or multiple targets, capable of revealing the presence of multiple reflection paths within the scenario while providing their respective time delays and reflection intensities [32]. This approach is well-suited to time-domain signal reconstruction in multipath scenarios, aligning closely with the research requirements of this study.

In urban environments, the channel frequency-domain response is obtained through the superposition of multipath coherence. Assuming the existence of M path propagation routes for electromagnetic waves, the frequency-domain response of all paths at frequency f can be expressed as:

(27) $$H\left(f\right)\;=\;\sum_{m\;=\;1}^{M}A_{m}\;e^{-j2\pi f\tau_{m}}$$

$A_{m}$ denotes the amplitude magnitude; $\tau_{m}$ denotes the propagation delay for different paths; *f* denotes the frequency.

In the linear time-invariant system, the impulse response h(t) can be obtained by performing IFFT on H(f) [30]:

(28) $$h(t)=F^{-1}\left\{H(f)\right\}=\sum_{m\;=\;1}^{M}A_{m}\delta (t\;-{\;\tau }_{m})$$

The formula for the N-point discrete IFFT is:

(29) $$h[n]\;=\;\frac{1}{N}\sum_{k\;=\;0}^{N-1}E[f_{k}]e^{j\frac{2\pi \mathrm{kn}}{N}},\;n\;=\;0,1,2,\ldots ,N-1$$

(30) $$f_{k}={\;f}_{0}+k\cdot \;\Delta \;f,\;k=0,1,2,\ldots ,N-1$$

where $f_{0}$ is the initial frequency; Δ f is the frequency difference; k is the index.

Substitution of Equation (27) into Equation (29) yields the following result:

(31) $$\begin{array}{c} h[n]\;=\;\frac{1}{N}\sum_{k\;=\;0}^{N-1}\left[\sum_{m\;=\;1}^{M}A_{m}e^{-j2\pi f\tau_{m}}\right]\cdot e^{j\frac{2\pi \mathrm{kn}}{N}}\\ =\sum_{m\;=\;1}^{M}A_{m}\left[\frac{1}{N}\sum_{k\;=\;0}^{N-1}e^{-j2\pi f\tau_{m}}\cdot e^{j\frac{2\pi \mathrm{kn}}{N}}\right] \end{array}$$

Substitution of Equation (30) into Equation (31) yields the following result:

(32) $$h[n]\;=\;\sum_{m\;=\;1}^{M}A_{m}e^{-j2\pi f_{0}\tau_{m}}\left[\frac{1}{N}\sum_{k\;=\;0}^{N-1}e^{j2\pi k\left(\frac{n}{N}-\Delta f\tau_{m}\right)}\right]$$

By utilizing the periodic sinc function to simplify the equation, the time-domain impulse response can be further derived:

(33) $$h[n]\;=\;\sum_{m\;=\;1}^{M}A_{m}e^{-j2\pi f_{0}\tau_{m}}\cdot \frac{\sin\left[\pi N\left(\frac{n}{N}-\Delta f\tau_{m}\right)\right]}{N\sin\left[\pi \left(\frac{n}{N}-\Delta f\tau_{m}\right)\right]}\cdot e^{j\pi (N-1)\left(\frac{n}{N}-\Delta f\tau_{m}\right)}$$

It can be demonstrated that the time-domain impulse response function can be further simplified to yield the following result:

(34) $$h[n]\;=\;\sum_{m\;=\;1}^{M}A_{m}e^{-j2\pi f_{0}\tau_{m}}\cdot {\sin c}_{N}\left(\frac{n}{N}-\Delta f\tau_{m}\right)$$

(35) $$\begin{array}{c} {\sin c}_{N}(x)=\frac{\sin(\pi \mathrm{Nx})}{N\sin(\pi x)}\cdot e^{j\pi (N-1)x}\\ x_{m}=\frac{n}{N}-\Delta f\tau_{m} \end{array}$$

The time corresponding to the time-domain sampling point is as follows:

(36) $$t_{n}\;=\;\frac{n}{N\cdot \Delta f},\;n\;=\;0,1,2,\ldots ,N-1$$

Time range:

(37) $$0\;\leq {\;t}_{n}\;<\;\frac{1}{\Delta f}$$

Time resolution is:

(38) $$\Delta t\;=\;\frac{1}{N\cdot \;\Delta f}$$

The time-domain impulse response waveform is obtained by performing an IFFT on the complex electric field at different frequencies. When the time-domain sampling points satisfy $\frac{n}{N\;\cdot \;\Delta f}\;\approx \;\tau_{m}$, the mth path generates a pulse with a peak amplitude approximately equal to $\left|A_{m}\right|$ on the corresponding time axis. It is possible to calculate the actual propagation distance of electromagnetic waves under multipath effects based on the different pulse delays.

It is evident that, given the time-domain signal comprises N sampling points within the time axis range and a sampling frequency interval of Δf, the reconstructed echo signal will exhibit a time resolution of $\frac{1}{N\cdot \Delta f}$, with a time axis range of $0\;\leq {\;t}_{n}\;<\;\frac{1}{\Delta f}$. Consequently, when executing the IFFT transformation for the frequency-domain response, it is imperative to guarantee a sufficient number of frequency sampling points. This enhancement in accuracy of electromagnetic simulation and the resolution of the echo signal is achieved while meeting the requirement for long-range radio detection that the reconstructed model’s time-axis range should not be excessively small. Consequently, for radio detection a distance of 1.5 km with a distance resolution of 20 m, the electromagnetic wave bandwidth must exceed 15 MHz, and the number of frequency sampling points must exceed 500. However, in practical IFFT transformations, factors such as phase discontinuities should be minimised. A broader frequency band provides more comprehensive information; however, an increase in bandwidth necessitates a greater number of frequency sampling points.

### 3.2. Pulse Signal Echo Simulation

The SBR method involves the utilization of electromagnetic waves within a designated frequency band for the scanning of the model. This process employs discrete frequency points within the band to perform single-frequency continuous wave simulations with identical amplitudes. In contrast to the continuous time-domain pulse signals commonly used in radar ranging, these simulations are performed with the discrete frequency points. In order to ascertain the equivalence of the reconstructed analogue signal post-SBR simulation with the radar pulse echo signal, a simulation of an example pulse echo signal is now conducted, with elevation angles of 30 and 90 degrees relative to the centre origin of the model, which is located 1.2 km from the model’s centre origin.

Construct a rectangular pulse signal *s*(*t*) with a center frequency of 6.1 GHz and a pulse width of 10 ns:

(39) $$s(t)\;=\;\mathrm{rect}(\frac{t-t_{0}}{T_{p}})\cdot \cos(2\pi f_{c}t)$$

where $T_{p}$ is the pulse width; $f_{c}$ is the carrier frequency; and $t_{0}$ is the delay.

**Figure 10:**
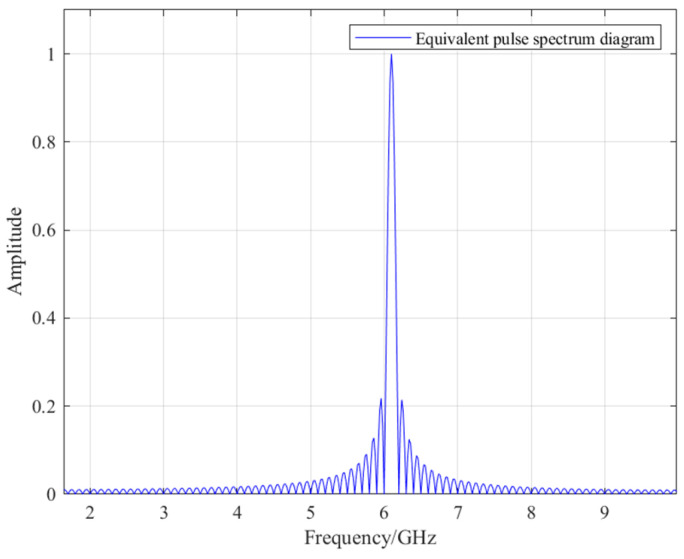
Spectrum diagram of rectangular pulse signal.

The spectrum diagram of *s*(*f*) is shown in Figure 10:

As shown in Figure 10, the pulse signal has a bandwidth of 200 MHz and a center frequency of 6.1 GHz.

The scanning parameters for electromagnetic simulation were set to the frequency band 6 GHz–6.2 GHz. In order to achieve equilibrium between temporal resolution and the time-axis range, 5000 sampling points were utilized to obtain the frequency-domain response *H(f)*.

The frequency domain response *H(f)* output from the SBR simulation is multiplied by the weighting function *W(f)*, thereby effectively transforming *H(f)* into the spectrum of the pulse signal s(t). Subsequently, an IFFT is applied to this equivalent pulse spectrum, thus completing the reconstruction of the time-domain pulse signal.

(40) $$P\left(f\right)=F\left\{s(t)\right\}$$

(41) $$R_{\mathrm{pulse}}\left(f\right)=H(f)\cdot w(f),\;w(f)=\left\{\begin{array}{ll} \frac{\left|P(f)\right|}{\left|H(f)\right|}, & f\in [6.0,\;6.2]\;\mathrm{GHz}\\ 0, & \mathrm{otherwise} \end{array}\right.$$

(42) $$R_{\mathrm{pulse}}\left(t\right)={\;F}^{-1}\left\{R_{\mathrm{pulse}}\left(f\right)\right\}$$

*H(f)* denotes the SBR simulation output value; P(f) represents the spectrum of the pulse signal; *w(f)* indicates the weighting factor at different frequency points; $R_{\mathrm{pulse}}\left(t\right)$ signifies the equivalent pulse spectrum; $R_{\mathrm{pulse}}\left(t\right)$ denotes the equivalent time-domain pulse signal; *F*$\left\{\cdot \right\}$ and $F^{-1}\left\{\cdot \right\}$ denote the Fourier transform and inverse Fourier transform, respectively.

**Figure 11:**
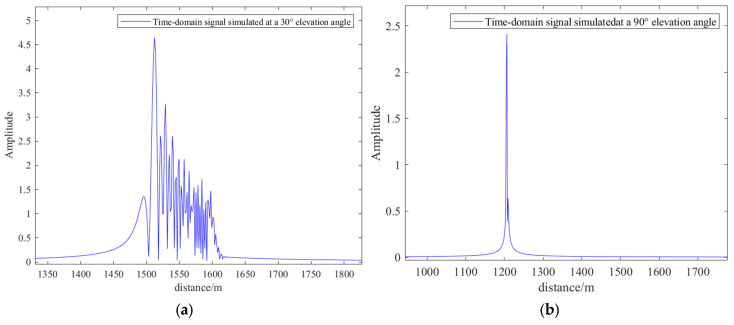
(**a**) Time-domain signal waveform at an elevation angle of 30 degrees and a distance of 1200 m from the model center. (**b**) Time-domain signal waveform at an elevation angle of 90 degrees and a distance of 1200 m from the model center.

Figure 10 generated the corresponding time-domain pulse diagram as shown in Figure 11:

Figure 11 reveals that the multipath propagation distances of simulated echo signals generated at a 30-degree elevation angle are primarily concentrated between 1500 m and 1600 m. The positions of different pulses along the horizontal axis represent propagation distances for distinct paths, while varying peak amplitudes reflect energy attenuation across different paths. This clearly demonstrates the multipath propagation of electromagnetic waves after multiple reflections within dense urban environments. At a 90 degree elevation angle, the echo signal exhibits only a single dominant peak. This reflects that when electromagnetic waves strike vertically, propagation is dominated by specular reflection, resulting in the shortest propagation distance.

## 4. Radio Altimeter Simulation

### 4.1. Radar Detection System Setup

The underlying principle of radar altimetry entails the precise measurement of the time interval for radar waves to travel from the platform to the ground surface and vice versa, thus facilitating the calculation of the distance between them. This facilitates the estimation of the vertical height, or slant range, from the radar antenna to the ground surface. The study under discussion herein designs a non-coherent pulse radar detection system, based on radio detection principles. The basic technical specifications of the radar system are shown in Table 3:

This study designed the radar detection system according to the parameters in Table 3. This study incorporates a range of factors into the radar ranging system, including environmental noise, thermal noise, atmospheric attenuation, and other interference factors. The purpose of incorporating these factors is to simulate a realistic urban environment. In order to counteract environmental interference while enhancing target detection accuracy, the radar system features pulse compression, time-varying gain (TVG) compensation, and constant false alarm rate (CFAR) adaptive threshold detection. This study uses vertical polarization for the simulation, which is the standard configuration for most commercial radio altimetry radars. The SBR method framework presented here is also based on linear polarisation. While circular polarization theoretically suppresses multipath signals from multiple reflections by rotating the polarization direction, it may be limited in altimetry applications. While circular polarization can suppress building multipath, it simultaneously attenuates the primary ground echo, which could degrade the signal-to-noise ratio. Nevertheless, using circular polarization to mitigate multipath reflection errors remains a viable approach. Future work will involve simulations and experiments with full polarization to accurately evaluate the effectiveness of circular polarization in suppressing multipath signals.

The operation of this system is primarily divided into three modules: the echo signal generation module, the signal processing and detection module, and the output module.

Echo signal generation module: The functionality of this module encompasses preprocessing operations, including down-conversion and low-pass filtering, on echo signals derived from electromagnetic simulation data. The purpose of these operations is to generate signals that are optimised for reception and subsequent processing by the radar system. The signal processing and detection module is a component of the system that is responsible for signal processing and detection. Firstly, matched filtering is applied in order to compress pulses and enhance the signal-to-noise ratio for echoes entering the radar system. Subsequently, signal propagation loss is compensated for via TVG compensation. Subsequently, the detection capability is enhanced through pulse accumulation. Ultimately, the system is able to detect targets by setting appropriate adaptive thresholds. Output module: The function in question produces a numerical value representing the quantity of targets that have been detected, whilst concurrently generating a time-domain signal plot. The ranging calculations are to be completed using the appropriate formula.

**Figure 12:**
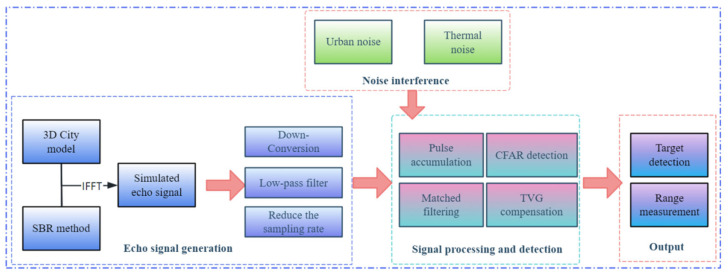
Radar System Workflow Diagram.

The basic workflow for radar system ranging is shown in Figure 12:

### 4.2. Simulated Echo Signal Input

Taking a simulated echo at an elevation angle of 30 degrees relative to the origin of Model A and a distance of 1200 m as an example, it is input into the radar detection system. The echo undergoes signal preprocessing, pulse compression, time-varying gain compensation, non-coherent accumulation, adaptive threshold detection, distance estimation, and other procedures to complete the full process of target height measurement.

**Figure 13:**
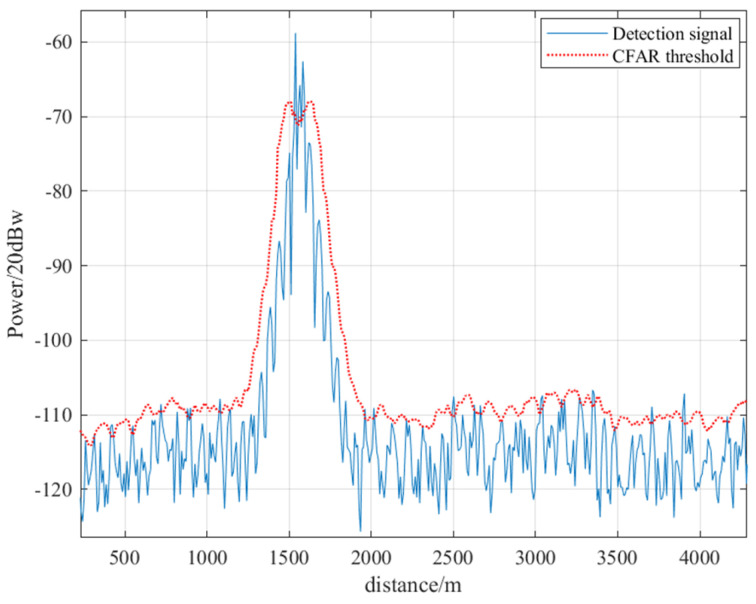
Input the simulated echo signal into the radar system to obtain the detection results.

As shown in Figure 13, after threshold detection, three peak signals were detected, with corresponding ranging results of 1552.50 m, 1575.00 m, and 1590.00 m. The ranging errors were 29.3%, 31.2%, and 32.5%, respectively.

### 4.3. Analysis of Distance Measurement Results

The echo simulation method and radio detection model described above were employed to perform simulated altimetry for urban Models A, B, and C at various elevation angles.

The ranging results for Model A are shown in Table 4:

The ranging results for Model B are shown in Table 5:

The ranging results for Model C are shown in Table 6:

This section of the study involved the completion of simulated elevation measurements for three distinct urban scenarios. The results obtained from the radar detection system demonstrate its ranging performance for simulated echoes at different elevation angles from a distance of 1200 metres. By comparing and analysing the data, we can systematically investigate the mechanisms by which multipath propagation of radar echoes in complex urban environments affects the accuracy of radar altimetry.

1. Low elevation angles cause multipath propagation to dominate ranging errors.

It is evident from the data presented in Table 4, Table 5 and Table 6 that electromagnetic waves undergo multiple reflections within the building complex. This phenomenon increases the propagation path length and results in significant delays in echo arrival times, consequently leading to ranging errors.

Secondly, it was determined that the incidence elevation angle had a significant influence on the propagation path of electromagnetic waves. At low elevation angles, an increase in the number of reflections was observed, accompanied by a lengthening of the propagation path. Multipath propagation effects became pronounced, and ranging errors were substantial. As the elevation angle increased, the number of reflections decreased, the path increment diminished, reflections approximated specular reflection, and multipath propagation was markedly suppressed. At an elevation angle of 90°, the errors for all three models remain below 2%, indicating that ranging is virtually unaffected by multipath effects. It can thus be concluded that propagation at low elevation angles is dominated by Non-line-of-sight conditions. In such cases, electromagnetic wave propagation is obstructed, leading to extensive reflection, diffraction, and scattering phenomena. These phenomena exacerbate ranging errors.

2. The Impact of Urban Geometric Characteristics on Multipath Propagation

As demonstrated in Table 4, Table 5 and Table 6, Model A demonstrates the largest ranging error at the same elevation angle, with Model B demonstrating the second-largest error and Model C demonstrating the smallest error. In the three-dimensional model under consideration, Model A exhibits the highest building density, with Model B ranking second and Model C third. The configuration of edifices in Model A is arranged in a regular grid pattern, causing rays to undergo multiple orderly reflections between walls, resulting in significant path increment accumulation. Model B exhibits a more chaotic arrangement of structures, with obstructions along certain pathways, which results in a reduced average path increment when compared to Model A. Model C features buildings with sparse layouts that are low-rise, and in this model, rays primarily undergo direct transmission or single reflections, resulting in minimal path increments. This demonstrates that increased building density enhances the number of electromagnetic wave reflections off walls, thereby extending the distance of multipath propagation.

3. Effect of building height on path increment

As demonstrated in Table 4, Table 5 and Table 6, and as evidenced by the geometric characteristics of the three models, it is apparent that ranging errors are more pronounced in scenarios involving tall buildings. Electromagnetic waves undergo multiple reflections within urban canyons, resulting in actual propagation distances that exceed direct line-of-sight distances. In an urban canyon of width *d*, the path increment is determined by the building height *H* and the elevation angle ε.

Maximum number of reflections *N* for rays within urban canyons:

(43) $$N\;\approx \;\frac{H}{d\tan\varepsilon }$$

Reflected path increment ∆l each time:

(44) $$∆l\;\approx \;\frac{d}{\cos\varepsilon }$$

Increment in total distance ΔL:

(45) $$\Delta L\propto \frac{H}{\sin\varepsilon }$$

As demonstrated in Formula (45), an increase in building height is associated with an increase in the propagation paths of electromagnetic waves.

A thorough investigation of the outcomes and electromagnetic simulation images reveals that in intricate urban settings, the presence of substantial structures and other objects results in the multifaceted propagation of electromagnetic waves. This process gives rise to the generation of additional propagation pathways, which in turn precipitate alterations in the primary peak during radar ranging, thus engendering substantial errors. Increased building density and height, in conjunction with reduced elevation angles for height measurement, serve to exacerbate this error.

The unique challenges inherent to kilometer-scale urban altimetry modelling have resulted in a paucity of publicly available benchmark datasets or standardized comparison methods. Consequently, this study does not undertake quantitative comparisons with alternative altimetry methods; future research will address this lacuna.

## 5. Conclusions

The construction of a kilometre-scale urban electromagnetic model and radar simulation system has been presented, along with a simulation of radio echoes in complex urban environments using the SBR method. The impact of complex urban environments on radio altimetry has also been investigated. A thoroughgoing analysis of electromagnetic simulation results and radar altimetry measurements across a range of scenarios has revealed that the presence of dense urban structures gives rise to multipath propagation of electromagnetic waves, resulting in substantial ranging errors. The aforementioned errors have been found to be correlated with building density, height, and the elevation angle of detection. In the context of radio altimetry applications in urban environments, sites characterised by relatively flat terrain, sparse building development, and high elevation angles should be accorded priority. The primary focus of this study is simulation modelling. Future work will emphasise experimental validation, incorporating the effects of Doppler shifts during motion, and examining differences in multipath energy accumulation among antennas with varying beam widths.

## Figures and Tables

**Table 1:** Path loss for different materials.

Material	Path Loss (dB)
Standard multipane glass	$L_{\mathrm{glass}}=2+0.2f$
Concrete	$L_{\mathrm{concrete}}=5+4f$
Plywood	$L_{\mathrm{plywood}}=1.03+0.17f$
Wood	$L_{\mathrm{wood}}=4.85+0.12f$

**Table 2:** Average RCS of the city model at different elevation angles.

Model	Elevation 10° RCS/dBsm	Elevation 30° RCS/dBsm	Elevation 50° RCS/dBsm	Elevation 70° RCS/dBsm	Elevation 90° RCS/dBsm
A	5.09	−0.34	−4.93	−1.46	24.36
B	7.36	2.24	7.45	6.57	28.92
C	−5.35	−3.086	−3.087	−1.02	34.98

**Table 3:** Basic technical specifications of the radar system.

Technical Specifications	Parameter
Maximum detection range	5 km
Probability of detection	90%
Probability of false alarm	$3\times 10^{-4}$
Range resolution	10 m
Threshold factor	1.8
Polarization mode	Vertical polarization

**Table 4:** At a distance of 1200 m from the center, the ranging results and error results of Model A at different elevation angles.

Scenario	Elevation Angle	Theoretical Distance/m	Measured Distance/m	Average Error
Model A	10°	1200	1552.50, 1575.00, 1579.50, 1612.50	32.05%
Model A	30°	1200	1552.50, 1575.00, 1590.00	31.00%
Model A	50°	1200	1328.00, 1344.00, 1368.00	12.20%
Model A	70°	1200	1384.00, 1408.00	16.50%
Model A	90°	1200	1177.50, 1200.00, 1215.00	1.03%

**Table 5:** At a distance of 1200 m from the center, the ranging results and error results of Model B at different elevation angles.

Scenario	Elevation Angle	Theoretical Distance/m	Measured Distance/m	Average Error
Model B	10°	1200	1408.00, 1456.00	19.30%
Model B	30°	1200	1432.00, 1448.00, 1464.00, 1488.00, 1520.00	22.54%
Model B	50°	1200	1320.00, 1360.00	11.65%
Model B	70°	1200	1216.00, 1248.00	2.65%
Model B	90°	1200	1200.00, 1240.00	1.70%

**Table 6:** At a distance of 1200 m from the center, the ranging results and error results of Model C at different elevation angles.

Scenario	Elevation Angle	Theoretical Distance/m	Measured Distance/m	Average Error
Model C	10°	1200	1170.00, 1190.00, 1220.00	1.64%
Model C	30°	1200	1210.00, 1243.00, 1265.00	3.24%
Model C	50°	1200	1212.00, 1236.00, 1272.00	3.30%
Model C	70°	1200	1200.80, 1216.60, 1240.30	1.62%
Model C	90°	1200	1192.00, 1208.00, 1232.00	1.30%

## Data Availability

The authors confirm that the data supporting the findings of this study are available within the article.

## Abbreviations

SBR	Shooting and Bouncing Rays
IFFT	Inverse Fast Fourier Transform
RCS	Radar Cross Section
FFT	Fast Fourier Transform
CFAR	Constant False Alarm Rate
GSM	Global System for Mobile Communications
3GPP	3rd Generation Partnership Project
ISAC	Integrated Sensing and Communication
TR-SAR	Time-Reverse Synthetic Aperture Radar
SAR	Synthetic Aperture Radar
GTD	Geometric Theory of Diffraction
UTD	Uniform Theory of Diffraction
GO	Geometric Optics
PO	Physical Optics
PEC	Perfect Electric Conductor
O2I	Outdoor-to-Indoor
TVG	Time-Varying Gain
MOM	Method of Moments
FDTD	Finite-Difference Time-Domain
LTI	Linear Time-Invariant
